# Assessing the economic impact of climate change in the small-scale aquaculture industry of Ghana, West Africa

**DOI:** 10.12688/aasopenres.12911.2

**Published:** 2019-10-17

**Authors:** Berchie Asiedu, Dickson Malcolm, Seidu Iddrisu

**Affiliations:** 1 Department of Fisheries and Water Resources, University of Energy and Natural Resources, Sunyani, Brong Ahafo, Ghana; 2 WorldFish Center, Dhaka, Bangladesh; 3 Water Research Institute, Water Research Institute, Tamale, Ghana; 4 Kocaeli University, Izmit-Kocaeli, Turkey

**Keywords:** aquaculture, climate change, impact, fish, profitability, and small-scale

## Abstract

**Background: **Aquaculture in Ghana is very profitable, but faces sustainability challenges. This paper assessed the impact pathways by which climate change affects the production and profitability of small-scale aquaculture in Ghana. The study analyzed and compared the economic value of smallholder fish farms with and without the incidence of climatic parameters.

**Methods: **Simple random sampling and purposive sampling techniques were used to select the study area and farms. A total of 30 farmers were interviewed using a questionnaire-based interview. Additionally, using document analysis, observation, and data on farms’ production input and output values, the economic impact of climate change on fish farms was assessed.

**Results: **Extreme temperatures, erratic rainfall, floods, drought, storm and erosion are prevalent in fish farms. Available data shows a decrease of 53.4% of small-scale revenue, a 6.9% reduction in small-scale aquaculture value from GH¢ 83,000 to GH¢ 120,000 reducing fish supply by 25%. The findings indicate that the profitability, economic value, and livelihoods of the small-scale aquaculture industry is greatly affected by changes in climate. The incidence of floods, drought, erratic rainfall, erosion, and extreme temperature synergistically induce poverty. The implication on the livelihoods of fish farming households is very alarming and poses a serious threat to food security in the country.

**Conclusion: **Based on the findings, this study concludes that; floods, rainfall temperature, and drought are the major climatic factors affecting the profitability and sustainability of the pond aquaculture industry. The preliminary recommendation is that there is an urgent need to map out flood-free zones close to perennial water bodies to overcome floods and droughts. Planting trees around ponds to create a micro-ecologies ideal for fish culture and also the construction of water storage facilities and proper dyke design would overcome drought and erosion issues. The adaptive capacity of fish-farmers must be built.

## Introduction

Food fish plays an important role in the livelihoods, nutrition, and security of millions of the people in Ghana and other parts of Africa. The small-scale aquaculture (SSA) sector, is recognized as making an important contribution to food and nutritional security, poverty alleviation, and socioeconomic development at the global level ( Bondad-Reantaso & Subasinghe, 2013; Sarker *et al*., 2017), especially in the developing countries. The global wild fish industry is failing, falling over the past decades from 85 million tonnes in 1995 to 80 million tonnes in 2014 ( FAO, 2016). Within the same period, the global per capita fish consumption has increased from 14 kg to 22 kg, which is credited to the growth in the aquaculture sector. The population of Africa is rising, with a corresponding increase in the demand for food, nutrition and livelihood support. While scientists and resource managers continue to battle with overfishing, pollution, and a decline in fish stocks, the impact of climate change is also attracting major attention. Variability in climate is modifying the productivity of aquatic ecosystems and thus affecting biological processes and food webs ( Yazdi & Shakouri, 2010). These impacts will be widely felt by fish farmers, fishers and the coastal poor through unstable livelihoods, fish availability and quality, and compromised health, safety, and homes ( De Silva & Soto, 2009; IPCC, 2007; and OECD, 2010). Many small-scale fish farmers live poorly due to low income level, low productivity, small pond size, low technology, and inadequate knowledge in aquaculture operations coupled with climate change. Climate change impacts like frequent floods, droughts, erosions, extreme temperature could worsen the situation of small-holder fish farming households through total stock loss, increased mortality, reduced fish yield, damage to ponds/tanks, increase operating cost, and affect their livelihoods ( Allison *et al*., 2005; De Silva & Soto, 2009; Yazdi & Shakouri, 2010).

Aquaculture development in Ghana is on the increase. Aquaculture currently contributes about 11% (52,470 mt) to annual fish production (465,357 mt) ( MoFAD, 2017). Pond-based aquaculture systems form about 35% of the production system, which is spread throughout all the regions in Ghana. However, many small pond-holder farmers have abandoned ponds due to floods, water scarcity and high mortality rate ( Asiedu *et al*., 2017; Hiheglo, 2008; Ragasa *et al*., 2018). But how the variability in climate affects the socio-economic status and livelihood of this already stressed and vulnerable group is not given the required attention. However, the population is growing (around 2.3% per annum and is projected to reach 37 million by 2030, GSS, 2014; GSS, 2017), poverty and hunger persist, unemployment is high, and fish demand is increasing.

Climate variability remains a threat to human settlement, and food production and supply systems for several decades. In recent years, the intensity of climate change and related impacts on the livelihoods of the poor and most vulnerable population has attracted the attention of scientists, researchers, governments, organizations, and other stakeholders. The incidence of floods, droughts, erratic rainfall, extreme temperature, and storm have become more prominent in most fish farming communities in Ghana ( Anane-Taabeah *et al*., 2010; Asiedu *et al*., 2017; Rurangwa *et al*., 2015). Ghana has recorded a 1°C rise in temperature over the past 6 decades ( Agyeman-Bonsu *et al*., 2008). The implication of this variation for food security and fish farmers’ livelihood is alarming. The consequential impacts could be direct or indirect on the socio-economic and livelihood of small-scale fish farming households. Smallholder farmers may have to incur extra costs to fight climate change shocks. In cases where farmers cannot cope with climatic losses, incidence such as flooding can carry their fish stocks away and, or damage their production systems, thereby becoming jobless and increasing their level of poverty. Accessing the cost of production, net revenue and cost farmers’ incurred to fight climate variability, promote aquaculture development and improve livelihood is therefore important.

In Ghana, the total national fish production remains below 50% of quantity demand forcing the Government to spend about US$ 131 million to import fish in 2016 ( MoFAD, 2017). The aquaculture subsector in country is underdeveloped, with the majority of farmers operating at the small-scale ( Asiedu *et al*., 2017; Kassam, 2014). Several studies have been carried out on aquaculture development in Ghana. The majority of emphasis has been on the cost of inputs, market systems, and infrastructure ( Kassam, 2014; Mboge, 2010; Rurangwa *et al*., 2015). The impacts of climate change on aquaculture is an area that is greatly underexploited. Available literature on climate change and aquaculture focus more on projections and predictions, not articulating properly the impacts of climate change at the farm level. This study goes beyond the art and science of predicting the potential implication of climate variability on aquaculture, but rather accessing and analyzing available data at the farm level to establish how climate change affects farmers’ livelihoods.

## Methods

### Study area

**Figure 1.**
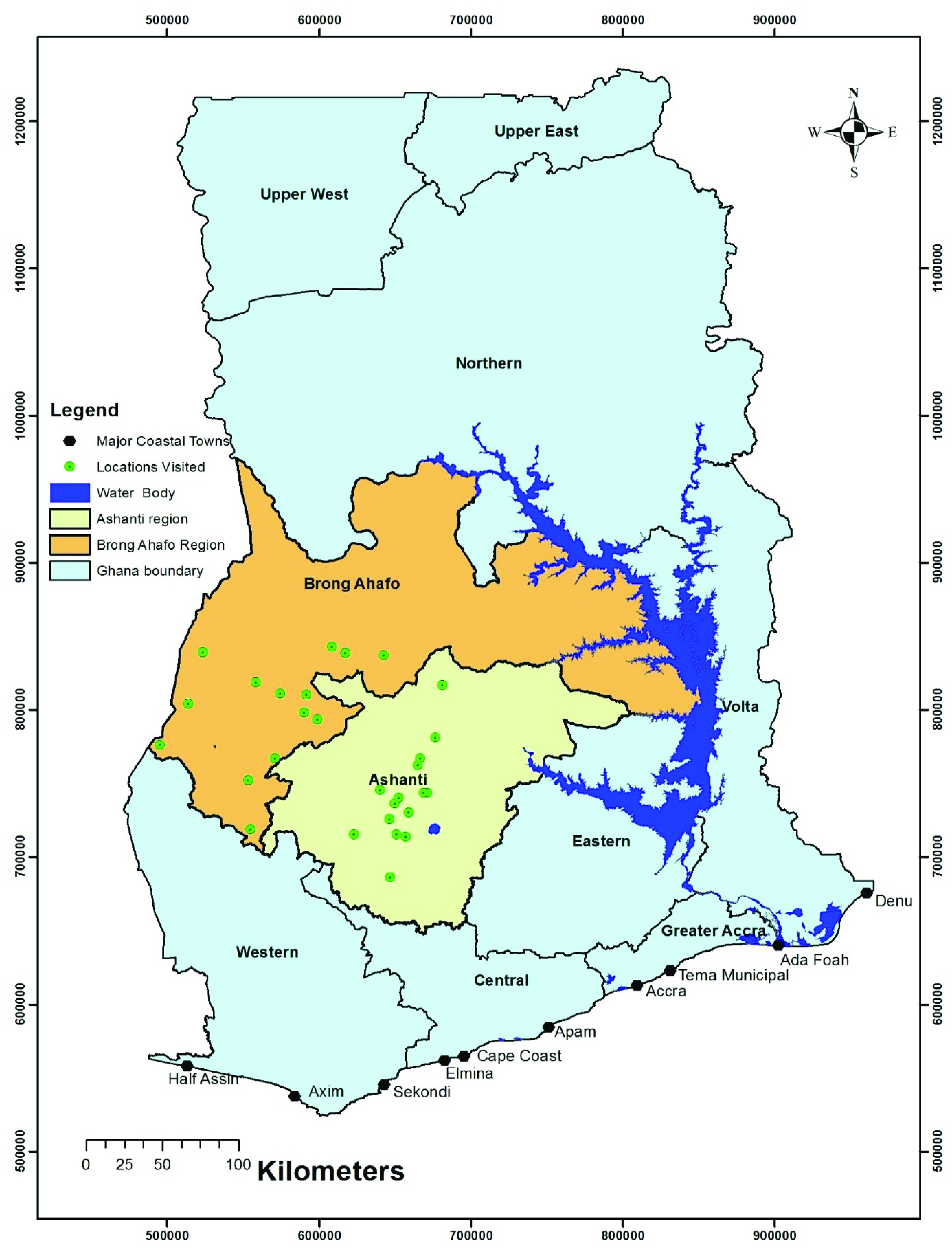
Map of Ghana showing locations visited for data collection.

The study was carried out in six fisheries zones in the Ashanti and Brong-Ahafo regions of Ghana ( Figure 1). The majority of small-scale aquaculture (ponds) in Ghana are concentrated in Brong-Ahafo and Ashanti regions. The two regions are the core of small-scale aquaculture practices, characterized by climatically favourable conditions, good market system, and a good environment. FSCBP (1997) indicated that the Ashanti region forms the highest potential area for aquaculture in Ghana. The Food and Agricultural Organization of the United Nations (FAO) (1991) mapped the region to be rich in the availability of land, water, rice bran and organic manure, thus suitable place for the viability of fish farming development. Generally, the whole area is inundated by rivers and streams which serve as a water source for fish farming ( Dankwa *et al*., 1999). The Ashanti region has about 1,200 fish ponds (Gyebi, personal communication).

The Brong-Ahafo region is endowed with good climate, rainfall system, and temperature ( MoFA, 2010). The region is a home to about 1,400 fish ponds, and 85% of its soil falls into the ochrosols groups, which generally have good water retention and are largely fertile ( MoFA, 2010). The topography is fairly flat, thus suitable for large scale pond construction. The region experiences a bimodal rainfall system which most farmers depend on to fill their ponds for their operations. The major rainy season is between March and September with the minor rainy season between October and December. This rainfall pattern offers two farming seasons in a year. These provide optimal conditions for farm fish production (See Figure 1).

### Sampling

The study employed a two-face sampling technique in site selection and data collection. A purposive sampling technique was first used to select two regions (Ashanti and Brong-Ahafo) out of ten regions in Ghana. The main target group was small-scale fish farmers, who are concentrated in the two regions. Fisheries zones were listed, and three randomly selected from each region. A list of registered farms with the Fisheries Commission was obtained, and simple random sampling used to select five farms from each fisheries zone for the farm survey. The random number feature of Microsoft Excel 2013 was used to randomly select fisheries zones and farms.

### Data collection

***Farmer interviews.*** A paper-based questionnaire guided interview was employed from June to December 2016. Key respondents for the farm level interview were either farmers or farm hand. The questionnaires (see Supplementary File 1 and Supplementary File 2) were structured to achieve the predetermined objectives of the study. Two sets of questionnaires were designed to target farm profitability and farm losses due to climatic effects. Farm profitability parameters included; cost of fingerling, cost of feed, cost of transportation, labour cost, quantity of fish harvested, and estimated value of harvest. Climatic factors herein refer to temperature, rainfall, floods, erosion, drought, and storms. These were modified from relevant studies ( Asiedu *et al*., 2017; De Silva & Soto 2009; Hiheglo, 2008; Mboge, 2010) of climatic change impacts on fisheries, aquaculture, agriculture, and livelihoods. The climatic factors were used to develop both quantitative and qualitative questions of open- and close-ended types. A total of 30 farm workers were interviewed, 15 in each region (Ashanti and Brong-Ahafo), each comprising 10 farmers and 5 farm hands. The interviews were conducted in an interactive manner, allowing farmers to ask questions and elaborate their points. This was to enable farmers to understand the significance of the study and to provide information relevant to the research.

***Desktop study.*** A desktop study was conducted. Notable reports, articles, and write-ups on climate change and related impacts on fisheries, aquaculture, agriculture and forestry were consulted. These included projected potential impacts of climate change on wild fisheries resources, cultured fisheries resources and on aquatic ecosystems. Keywords used included: food security, climate change, climate controls, climate change and food systems, climate change and aquaculture in the developing world, climate change implications for fisheries and aquaculture. Climatic factors identified impacting fisheries and aquaculture greatly through direct and indirect impact pathways were used to modify qualitative and quantitative questions for the field data collection. The following online resources were utilized: FAO GeoNetwork; FAO Climpag, FAO GIEWS, WorldFish Center, CGIAR Research Program on Climate Change, Agriculture and Food Security, The Technical Centre for Agricultural Research (CTA), Organisation for Economic Co-operation and Development (OECD) and the World Farmers’ Organisation (WFO). The results of the desktop study are presented in the Introduction and Discussion sections.

***Validation workshop.*** A one-day validation workshop was organised for farmers, fisheries administration officials, NGOs, regional organizations, academia/researchers to validate the findings collected during field data collection.

***Ethics and consent.*** The study received ethical approval from the University of Energy and Natural Resources Research, Conferences and Scholarships Committee, and ensured informed consent was received for data collection and analysis. Three key ethical issues considered were: voluntary participation; anonymity and confidentiality with respect to data analysis in collective manner were ensured. Furthermore during the validation workshop, the participants were openly told of the purpose of the workshop and consent was sought verbally. Verbal consent was sought as the farmers were unable to read or write.

### Data analysis

Quantitative and qualitative data collected during the field study included: a) operating cost: feed, fingerling, transport, harvest, and labour, b) outputs: total fish weight and value, and c) the incidence of floods, storms, temperature, droughts, rainfall, and erosion and their impacts on farm revenue and maintenance. All completed data collected was manually entered into Microsoft Excel 2013 and SPSS version 22 to await analysis ( Asiedu, 2018). The results from the analyzed data is presented in the form of tables, and charts/graphs. Observation data was used as a guide to validate farmer’s description of climatic factors. These include: topography, water source, pond size, evidence of erosion, local strategies put in place to mitigate impacts.

### Estimations and assumptions

- Total production cost ( *tpc*): the sum total of variable inputs example feed, fingerlings, labour, water, weed control, transport, and others as stated by the farmer. This is given by; $tpc=\sum (x_{1},\;x_{2}\cdots \;\cdots \;\cdots \;\cdots \;x_{n}),x=\text{variable}\;\;\text{cost}.$
- Total wet weight (TWW): is the product of average weight ( *aw*) of fish at the time of sales and the total number of fish harvested ( *nfh*). This is estimated using; tww = aw*nfh
- Estimated value of harvest ( *evh*): is the product of the unit market price of fish (cost ^-kg^) and the total units harvested (tww). Thus: *evh =* $cost^{-kg}*tww$
- Non-climate/net revenue: defined as the estimated value minus total production cost. It is given by the relation:
  $$nonclimate\;\;revenue=\text{(}cost^{-kg}\;\text{*}\;tww\text{)}-\sum \text{(}x_{\text{1}}\cdots \;\cdot x_{n}\text{)}$$

## Results and discussion

### Impact of climate change on aquaculture revenue

**Figure 2.**
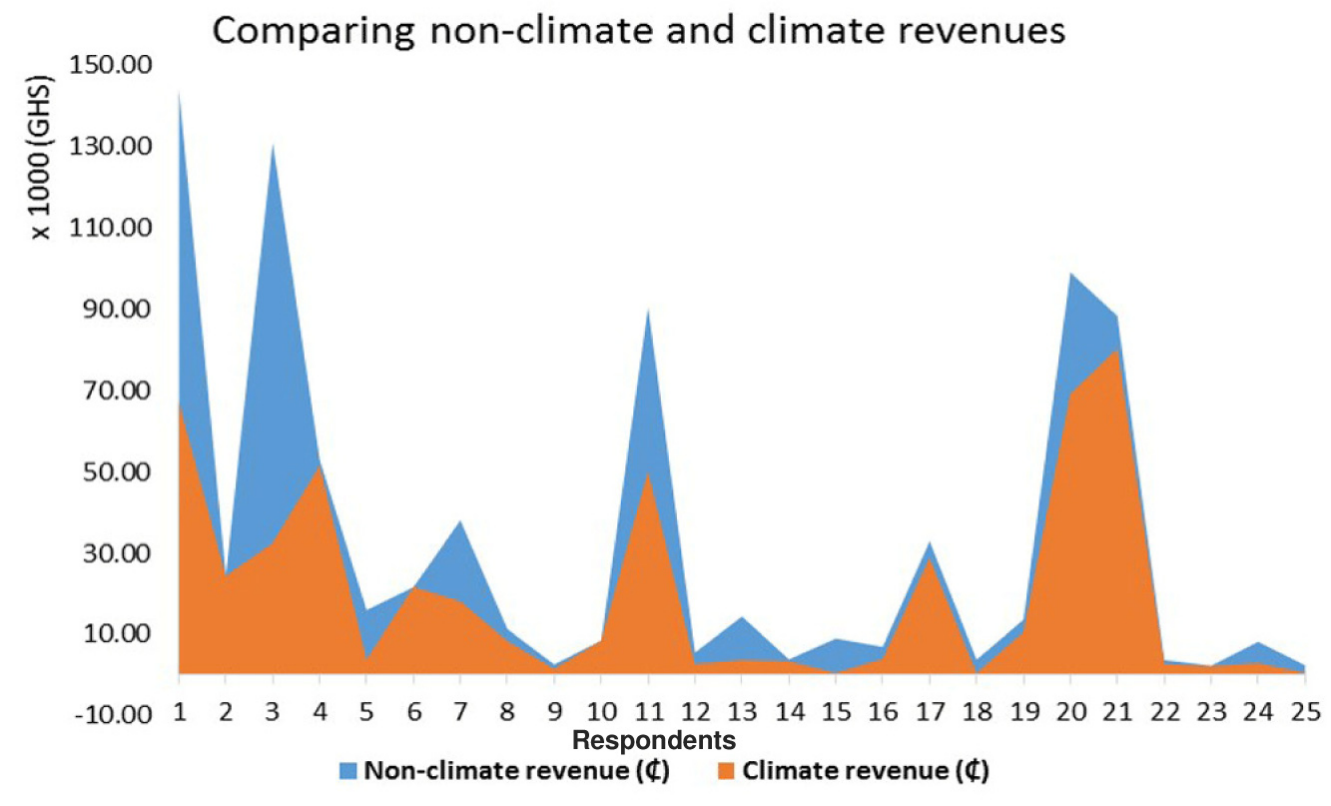
Impact of climate change on small-scale aquaculture revenue.

Farm revenue is a key profitability indicator of a project. Figure 2 compares non-climate revenues and climate revenues. In this study, non-climate revenue is the difference between the value of fish harvested (total cash inflows) and total production cost (total cash outflows). Whilst climate revenue is the difference between non-climate revenue and total cost climatic impact. Non-climate revenue is also referred to as actual revenue in the study.

Climate change poses an alarming threat to the economic development of the small-scale aquaculture industry in Ghana. This is evident in the gap between non-climate revenue and climate revenue as presented in Figure 2. The highest non-climate revenue stands at GH₵ 144,130 with a production cost of GH₵ 95,870 at point 1. This is about 50.3% of the amount invested. Hiheglo (2008) and Mboge (2010) predicted that small-scale aquaculture could generate a profit of 77% and 72%, respectively. This is a confirmation of the suggestion of Kassam (2014) that there may be an asset threshold over which fish farming allows higher income and asset accumulation than non-fish farming households. The climate revenue recorded at point 1 is GH₵ 67,130. This represents 53.4% loss of the farmer’s non-climate revenue, over half of the farmers’ economic returns. OECD (2010) noted that the socio-economic effects of climate change on fisheries and aquaculture are hard to determine but could amount to many billions of dollars. Comparatively, the climate total revenue is 40.2% of the non-climate revenue. The severity of climate change impact on aquaculture will depend on farm location, cultured fish species, and water used ( Asiedu *et al*., 2017; Hiheglo, 2008; Poff *et al*., 2002).

The incidence of floods, extreme temperature, erratic rainfalls, droughts, and erosion are causing significant economic loss to small-scale fish farmers. Direct effects may be through stock loss, damage of infrastructure, high rate of mortality, and growth altering, water scarcity, and reproductive capacity ( De Silva & Soto, 2009; Handisyde *et al.*, 2006; Yazdi & Shakouri, 2010), and thus affecting farming production and profit. Asiedu *et al*. (2017) indicated that changes in weather patterns and related disasters have led to serious economic losses in fish farms, loss of fish stock to floods, droughts, and damage of pond dike by erosion. But the study did not quantify the economic losses due to climate impacts.

### Small-scale aquaculture non-climate value and climate value

**Figure 3.**
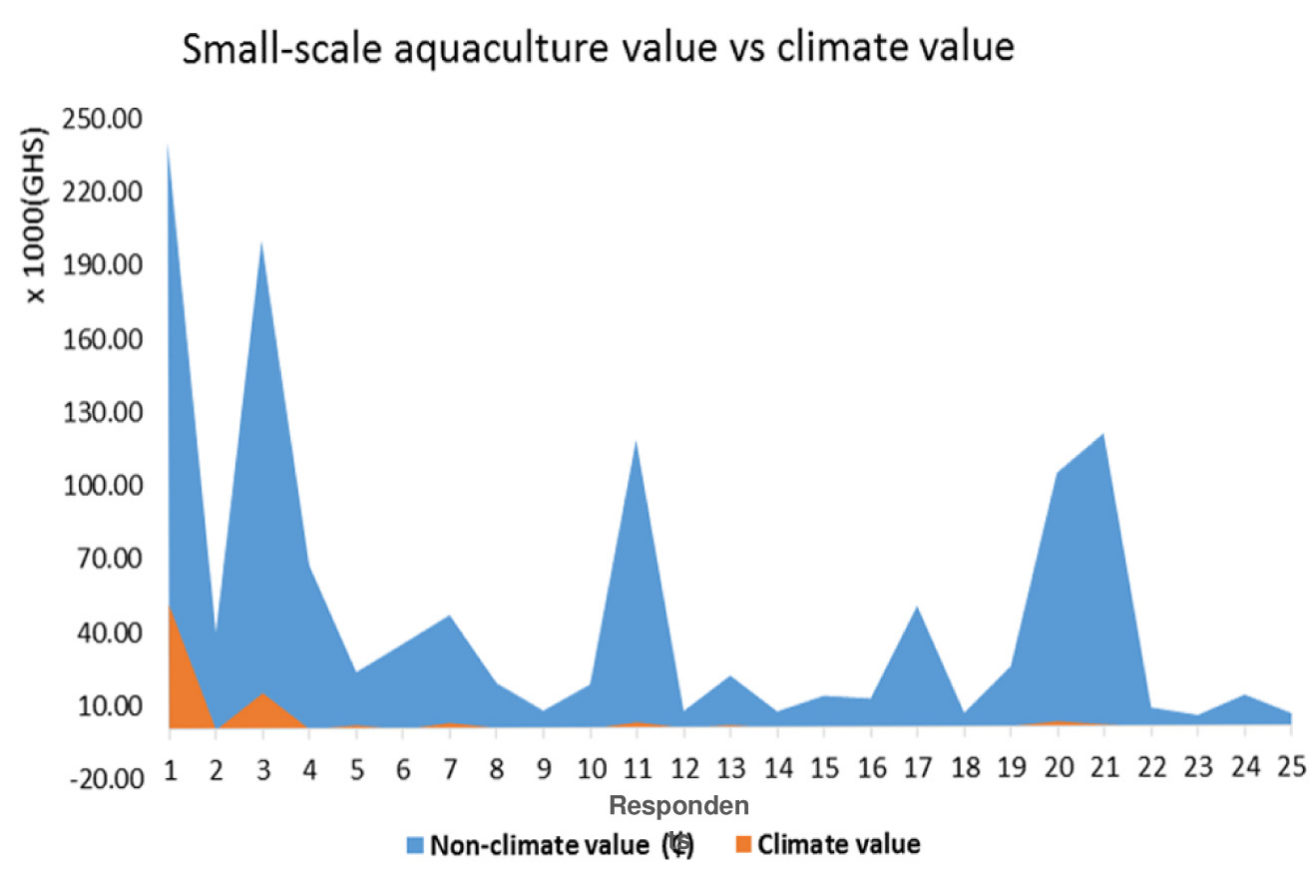
Impact of climate change on small-scale aquaculture yield.

The value of fish of an aquaculture establishment gives a telling detail of its ability to improve the socio-economic and livelihood of fish farming households. Value estimation is an important economic parameter informing the decision of investment. The non-climate value of small-scale aquaculture and climate value are compared in Figure 3 below. The total value in this study stands at over GH₵ 1.2 million for an estimated wet weight of 84.4 metric tonnes ( Supplementary Table 1). This proves why small-scale aquaculture is widely considered as economically viable venture worth million tons and billions of dollars, and improving the lives of people ( Bondad-Reantaso & Subasinghe, 2013; FAO, 2015; Worldfish, 2010). This is sustainable in the case of climate change effects and resultant impact pathways. Climate change impacts the economic value of small-scale aquaculture drastically, by reducing non-climate value of GH₵1.2 million to GH₵ 83,191.98 ( Supplementary Table 1). This represents 6.9% reduction in small-scale aquaculture value. De Silva & Soto (2009) noted that changes in water availability, extreme weather events, vertical stratification, and nutrient supply may have negative effects on freshwater aquaculture production, relative to local conditions. This is observable in Figure 3 below, where climate value is nearly invisible and farmers do not incur many losses to climatic effects.

### Climate change, food and nutrition security

**Figure 4.**
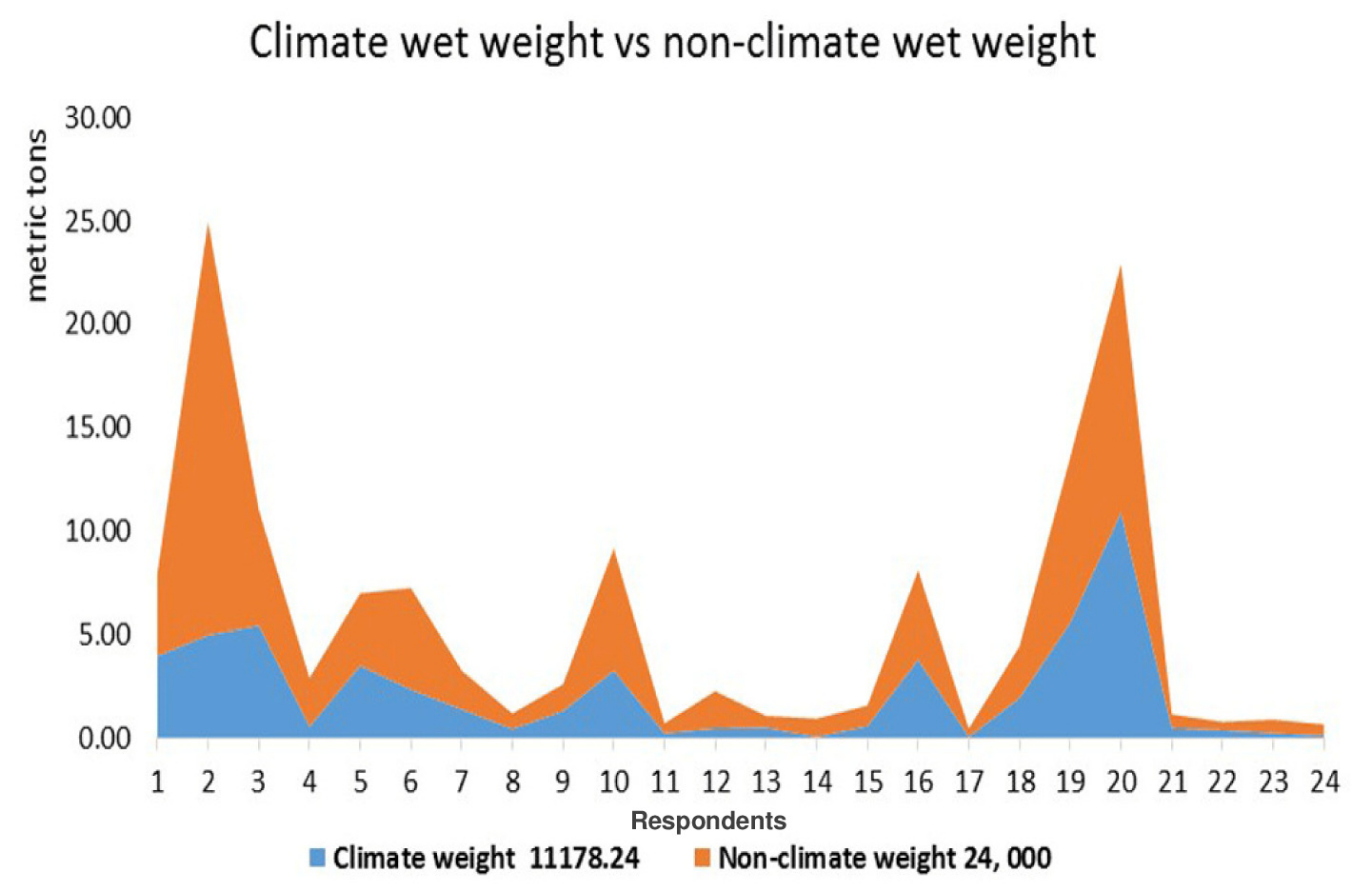
Impact of climate change on aquaculture production.

The small-scale fish farming industry contributes significantly to food and nutrition security in Ghana, thus promoting the “blue revolution” concept and ensuring sustainable development. A single pond is capable of producing 24.0 mt/cycle ( Figure 4). The total estimated small-scale production of sampled farms was 84.4 metric tonnes (mt) ( Supplementary Table 1). Considering the estimated per capita fish consumption of 26 kg (national average), these sampled ponds have the potential to providing food to over 3,000 people, thereby providing more balanced diets and better nutrition ( Golden *et al*., 2017; Toufique & Belton, 2014; Troell *et al*., 2014). Worldfish (2011) stated that millions of poor people in the developing countries of Africa and Asia rely on a combination of fishing and farming to earn their livelihoods and feed their families. But variation in climatic conditions is changing the trend. Analyzed data indicate about 25% reduction in small-scale aquaculture production from 84.4 mt ( Supplementary Table 1) to 63.6 mt ( Supplementary Table 2) due to incidence of floods, extreme temperatures, droughts, and erosion. Comparatively, the climate wet weight remains lower than the non-climate wet weight throughout the farms assessed (see Figure 4). This threatens the nutrition security and livelihoods of the rural poor fish farmers. Yazdi & Shakouri (2010) noted that the impact of environmental variability could affect the food and water scarcity of many people in the world by affecting fish production and the socioeconomic livelihood of already stressed small-scale fisheries communities.

### Incidence of climatic disasters and costs

**Figure 5.**
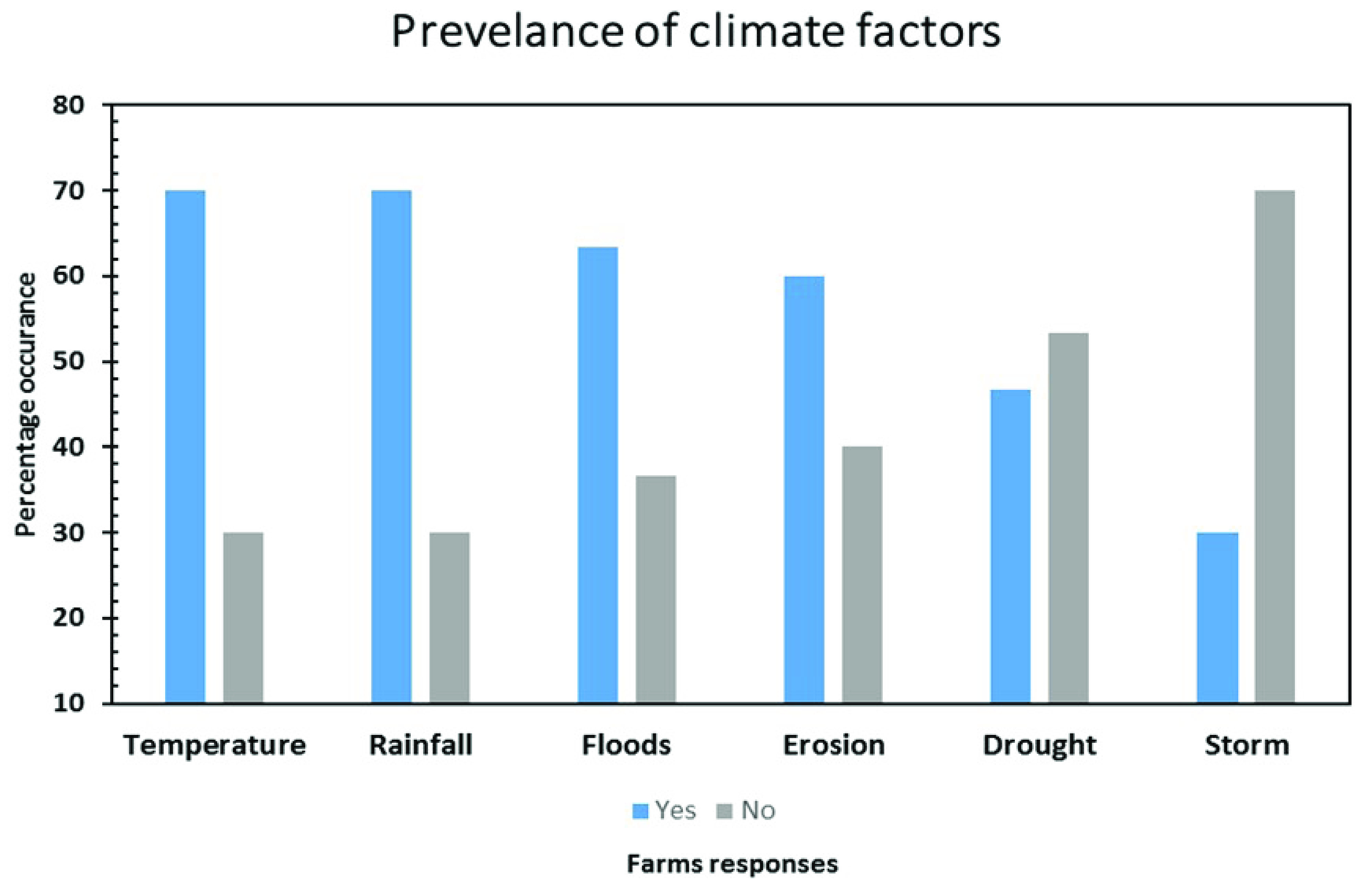
Incidence of climate change variable at fish farms.

The occurrence of climate related disasters at fish farms is presented in Figure 5. Climate related disasters in this context refer to climatic factors; flood, temperature, rainfall, drought, and erosion. Extreme temperature and erratic rainfall are the climatic factors mostly experienced by smallholder fish farmers at the same level, 70% (21, *n=*30) for each. The incidence of flooding and pond erosion were also relatively high, contributing 63.3% and 60%, respectively. Whilst some 46.7% of farms experienced droughts and 30% also experienced storms ( Figure 5). Mboge (2010) noted that flooding and erosion were the most prevalent natural disasters affecting pond aquaculture sustainability. This can be attributed to variability in environmental patterns resulting in changing disaster incidences. De Silva & Soto (2009) noted that temperature and salinity may impact aquaculture positively or negatively. Changes in temperature patterns affect the formation of clouds and rainfall pattern. This could result in either extremely low rains (inadequate water supply and droughts) or torrential rains that may result in serious floods and erosion of ponds. Changes in rain pattern will affect water availability ranging from droughts and shortages to floods and will reduce water quality, and threaten inland freshwater aquaculture ( IPCC, 2007). Similarly increasing temperature affects dissolved oxygen and increase fish metabolism, heightens fish deaths, drops in production or increases in feed requirements while also increasing the risk and spread of disease ( Allison *et al*., 2005; FAO, 2008; IPCC, 2007). This may probably account for the recognition of rainfall, temperature, floods, and erosion as the most prevalent climatic factors causing significant economic losses on pond fish farms (see Figure 5).

**Figure 6.**
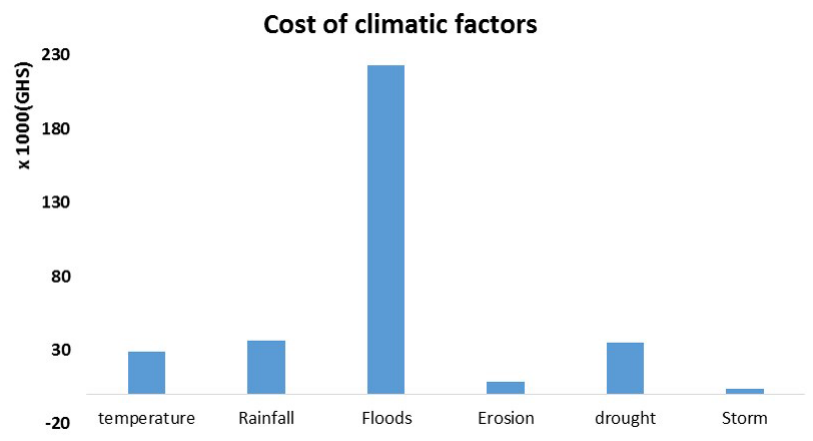
Climate related disasters and cost on farm value.

Although extreme temperature and erratic rainfall are the most prevalent climate disasters, flood is the most dominant climatic factor of significant economic impact on small-scale aquaculture ( Figure 6). Flood stimulates poverty by reducing farmers’ profitability significantly. This will hinder the effort of using aquaculture to sustainably reduce levels of poverty and improve economic performance in the country. Evidently, the total cost of flood impact in this study is estimated stands at GH₵ 223,435. This makes up 26.7% of the total non-climate revenue. The climatic factors with minimal economic impact in this study are storms (GH₵ 3,650) and erosion (GH₵ 8,210). OECD (2010) noted that typhoon-induced floods have had major impacts on the aquaculture industry through facilities damage and escapes of cultured fish. Floods are particularly disastrous for the world’s poor, causing estimated agriculture losses of USD 5.1 billion ( FAO, 2018). The most prevalent climatic factors, rainfall and temperature, also cause significant impact worth GH₵ 36,450 and GH₵ 29,195, respectively. The total cost of climate impact is estimated at GH₵ 336,230, which is 40.2% of non-climate revenue.

## Conclusion

This study was carried out with an emphasis on aquaculture sustainability. The problem of food and nutrition insecurity still persists. Undoubtedly, the population of Ghana is growing rapidly. At the same time, there is increasing pressure on food production and supply systems due to the growing demand for food and increasing purchasing power. The major focus now is not only feeding the population but also on environmental safety and health. It is evident from the study that the changing climate has caused a serious reduction in small-scale aquaculture profitability. The aspect of climate change that will be detrimental to aquaculture growth is torrential rains-induced floods. The research showed that climate change will compromise the profitability and sustainability of pond aquaculture through affecting the physical structure (ponds and tanks) of farms and the loss of fish stocks. The likely and most obvious outcome will be increasing poverty, food insecurity, and malnutrition. Farmers will be compelled to abandon ponds, stop production and become jobless if this trend continues, or adapt to the changes. This will not only decrease food and protein availability, but hinders the global goal to “end poverty, in all forms, everywhere” ( UN, 2018). Based on the findings, this study concludes that floods, rainfall temperature, and drought are the major climatic factors affecting the profitability and sustainability of the pond aquaculture industry. The preliminary recommendation is that there is an urgent need to map out flood-free zones close to perennial water bodies to overcome floods and droughts. Planting trees around ponds to create a micro-ecologies ideal for fish culture; additionally, the construction of water storage facilities and proper dyke design would overcome drought and erosion issues. Furthermore, the Ministry of Fisheries and Aquaculture Development should urgently develop a national policy on climate change and aquaculture, as well as establish climate change and fisheries/aquaculture unit to monitor and document all climate change issues.

## Data availability

The raw data associated with this study are available on OSF. DOI: https://dx.doi.org/10.17605/OSF.IO/B2KP4 ( Asiedu, 2018). Data are available under the terms of the Creative Commons Zero "No rights reserved" data waiver (CC0 1.0 Public domain dedication).

## Supplementary material

**Supplementary File 1. Questionnaire concerning the economics and profitability of aquaculture in Ghana.**

**Supplementary File 2. Questionnaire concerning the impact of climate change at farm and community levels.**

**Supplementary Table 1. Production and profitability of pond aquaculture.**

**Supplementary Table 2. Incidence of climatic disasters and attributed costs.**

**Supplementary Table 3. Analyzed climate impact areas.**
