# Supplementary material for: Assessing the economic impact of climate change in the small-scale aquaculture industry of Ghana, West Africa
Source: AAS Open Res. 2019 Oct 17;1:26. Originally published 2018 Nov 1. [Version 2] doi: 10.12688/aasopenres.12911.2 (PMC7391010; doi:10.12688/aasopenres.12911.2)
Supplement: Supplementary file 1 [file aasopenres-1-14095-s0000.tgz › 135fd7c0-9e14-4d47-9c39-a6e65982edcd_Questionnaire_1.docx]

**Supplementary File 1:** Economic and profitability of aquaculture in Ghana

**CLIMATE IMPACTS RESEARCH CAPACITY LEADERSHIP ENHANCEMENT (CIRCLE) PROGRAMME**

**Farm name…………………........................**

**Condition……………………………...…...**

**Date…………………………………………**

**Farm ID……………………………………**

**Location…....………………………………**

**Number of ponds………….……………….**

Aquaculture and Economic data:

1. Field measurement of water quality parameters (samples are taken from three ponds for measurement)

| Pond ID | Dissolved oxygen (mg/L) | Temperature (ºC) | Turbidity | pH |
| --- | --- | --- | --- | --- |
| A |  |  |  |  |
| B |  |  |  |  |
| C |  |  |  |  |

| Unit ID | Area (m^2^) | Stocking cost (GH¢) | Feeding cost (GH¢) | Other* costs (GH¢) | Price of harvested (GH¢) | Total production cost (GH¢) |
| --- | --- | --- | --- | --- | --- | --- |
| 1 |  |  |  |  |  |  |
| 2 |  |  |  |  |  |  |
| 3 |  |  |  |  |  |  |
| 4 |  |  |  |  |  |  |
| 5 |  |  |  |  |  |  |
| 6 |  |  |  |  |  |  |
| 7 |  |  |  |  |  |  |

1. Indicate in the table below your production cost per production unit for one production cycle, stocking, costs and price should be based on averages.

| Unit ID | Fish species | Farming technology | Cost per kg (GH¢/kg) | Wet weight (kg) |
| --- | --- | --- | --- | --- |
| 1 |  |  |  |  |
| 2 |  |  |  |  |
| 3 |  |  |  |  |
| 4 |  |  |  |  |
| 5 |  |  |  |  |
| 6 |  |  |  |  |
| 7 |  |  |  |  |
| 8 |  |  |  |  |

1. How much quantity of fish (in kg) by production unit do you produce in a cycle? Please indicate in the table below;

| Source of water | Adequate? Y/N | Estimate quality | Treatment before use |
| --- | --- | --- | --- |
| Stream |  |  |  |
| River basin |  |  |  |
| Rainfall |  |  |  |
| Underground |  |  |  |
| Wells |  |  |  |

1. Please indicate your water source, quality and treatment before use
