# Supplementary material for: Assessing the economic impact of climate change in the small-scale aquaculture industry of Ghana, West Africa
Source: AAS Open Res. 2019 Oct 17;1:26. Originally published 2018 Nov 1. [Version 2] doi: 10.12688/aasopenres.12911.2 (PMC7391010; doi:10.12688/aasopenres.12911.2)
Supplement: Supplementary file 2 [file aasopenres-1-14095-s0001.tgz › edb6432b-a0f5-488e-8460-67d6c1990023_Questionnaire_2.docx]

**Supplementary File 2:** Impact of climate change at Farm and Community Levels (6months-1year)

**CLIMATE IMPACTS RESEARCH CAPACITY LEADERSHIP ENHANCEMENT (CIRCLE) PROGRAMME**

Name: Contact:

Time: Location:

Impact of climate change at Farm and Community Levels (6months-1year)

| Climatic factor | Experience at: | | | | Period normally experience | Effects on: | |
| --- | --- | --- | --- | --- | --- | --- | --- |
|  | Farm (past 6 months) | Community (past 6 months) | Farm (past 1yr.) | Community (past 1yr.) |  | Farm | Community |
| Rising temperature |  |  |  |  |  |  |  |
| Change in rainfall pattern |  |  |  |  |  |  |  |
| Drought |  |  |  |  |  |  |  |
| Flood |  |  |  |  |  |  |  |
| Strong winds |  |  |  |  |  |  |  |
| Weather related disasters |  |  |  |  |  |  |  |
