# Supplementary material for: Assessing the economic impact of climate change in the small-scale aquaculture industry of Ghana, West Africa
Source: AAS Open Res. 2019 Oct 17;1:26. Originally published 2018 Nov 1. [Version 2] doi: 10.12688/aasopenres.12911.2 (PMC7391010; doi:10.12688/aasopenres.12911.2)
Supplement: Supplementary file 3 [file aasopenres-1-14095-s0002.tgz › ab762dd6-bb6b-432f-8455-abd423327b15_Supplementary_Table_1.docx]

| **Supplementary Table 1: Production and Profitability of Pond Aquaculture** | | | | | | | |
| --- | --- | --- | --- | --- | --- | --- | --- |
| Farm | surface area (m^2^) | Species cultured | Farming technology | Total Production Cost (₵) | Total Wet Weight(kg) | Total Value Harvest (₵) | Revenue (₵) |
| 1 | 7380.00 | O. niloticus | Monoculture | 95,870 | 24, 000 | 240,000.00 | 144,130.00 |
| 2 | 390.00 | C. gariepinus | Monoculture | 15,460 | 4000 | 40,000.00 | 24,540.00 |
| 3 | 4200.00 | C. O. & H. | Polyculture | 69,000 | 20000 | 200,000.00 | 131,000.00 |
| 4 | 1640.00 | C. & O. | Polyculture | 14,156 | 5600 | 67,200.00 | 53,044.00 |
| 5 | 2450.00 | C. & O. | Polyculture | 7,497 | 2346.5 | 23,465.00 | 15,968.00 |
| 6 | 1200.00 | O. & C. | Polyculture | 13,350 | 3500 | 35,000.00 | 21,650.00 |
| 7 | 735.50 | O. & C. | Polyculture | 8,620 | 4915.3 | 46,695.35 | 38,075.00 |
| 8 | 780.00 | O. & C. | Polyculture | 7,460 | 1880 | 18,800.00 | 11,340.00 |
| 9 | . | C. gariepinus | Monoculture | 4,900 | 750 | 7,500.00 | 2,600.00 |
| 10 | 700.00 | C. gariepinus | Monoculture | 9,700 | 1300 | 18,200.00 | 8,500.00 |
| 11 | . | C. gariepinus | Monoculture | 27,580 | 5900 | 118,000.00 | 90,420.00 |
| 12 | 300.00 | O. niloticus | Monoculture | 1,560 | 474 | 7,110.00 | 5,550.00 |
| 13 | 900.00 | O&C | Polyculture | 7,130 | 1795 | 21,540.00 | 14,410.00 |
| 14 | 618.00 | O. niloticus | Monoculture | 2,974 | 570 | 6,840.00 | 3,866.00 |
| 15 | 1100.00 | O. niloticus | Monoculture | 4,203 | 880 | 13,200.00 | 8,997.00 |
| 16 | 1800.00 | O. niloticus | Monoculture | 5,040 | 1000 | 11,999.99 | 6,959.99 |
| 17 | 2700.00 | O. niloticus | Monoculture | 16,860 | 4320 | 49,800.00 | 32,940.00 |
| 18 | 1200.00 | O. niloticus | Monoculture | 2,200 | 400 | 6,000.00 | 3,800.00 |
| 19 | 600.00 | C. gariepinus | Monoculture | 11,370 | 2500 | 25,000.00 | 13,630.00 |
| 20 | 900.00 | O. niloticus | Monoculture | 4,895 | 8000 | 104,000.00 | 99,105.00 |
| 21 | 1360.00 | C. gariepinus | Monoculture | 31,620 | 12000 | 120,000.00 | 88,380.00 |
| 22 | 341.00 | O. niloticus | Monoculture | 4,164 | 656 | 7,872.00 | 3,708.00 |
| 23 | 600.00 | O. niloticus | Monoculture | 2,500 | 400 | 4,800.00 | 2,300.00 |
| 24 | 450.00 | C. gariepinus | Monoculture | 4,800 | 650 | 13,000.00 | 8,200.00 |
| 25 | 590.00 | O. niloticus | Monoculture | 3,000 | 540 | 5,400.00 | 2,400.00 |
|  |  |  |  |  |  |  |  |
| **Total** |  |  |  | **280,039.00** | **84,376.80** | **1,211,422.34** | **835,512.99** |
