# Supplementary material for: Assessing the economic impact of climate change in the small-scale aquaculture industry of Ghana, West Africa
Source: AAS Open Res. 2019 Oct 17;1:26. Originally published 2018 Nov 1. [Version 2] doi: 10.12688/aasopenres.12911.2 (PMC7391010; doi:10.12688/aasopenres.12911.2)
Supplement: Supplementary file 4 [file aasopenres-1-14095-s0003.tgz › 93f85ef1-08eb-49ee-a921-d9a152389b76_Supplementary_Table_2.docx]

**Supplementary Table 2: incidence of climatic disasters and attributed costs**

| **Farm** | **Temperature (**GH₵) | **Rainfall**  **(**GH₵) | **Floods (**GH₵) | **Erosion (**GH₵) | **Drought (**GH₵) | **Storm (**GH₵) |
| --- | --- | --- | --- | --- | --- | --- |
| 1 | 0 | 0 | 50000 | 2000 | 25000 | 0 |
| 2 | 0 | 0 | 0 | 200 | 0 | 0 |
| 3 | 20000 | 0 | 75000 | 0 | 3500 | 0 |
| 4 | 0 | 0 | 0 | 1500 | 0 | 0 |
| 5 | 200 | 0 | 12000 | 0 | 0 | 0 |
| 6 | 0 | 0 | 0 | 0 | 0 | 0 |
| 7 | 2500 | 1830 | 15000 | 600 | 0 | 0 |
| 8 | 0 | 3000 | 0 | 0 | 0 | 0 |
| 9 | 0 | 0 | 700 | 400 | 0 | 0 |
| 10 | 0 | 0 | 15 | 0 | 0 | 0 |
| 11 | 240 | 20000 | 20000 | 100 | 0 | 50 |
| 12 | 0 | 2400 | 0 | 0 | 500 | 0 |
| 13 | 500 | 200 | 10000 | 0 |  | 0 |
| 14 | 200 | 50 | 0 | 240 | 0 | 0 |
| 15 | 1500 | 3000 | 4000 | 0 | 0 | 0 |
| 16 | 0 | 0 | 2000 | 0 | 1000 | 0 |
| 17 | 2000 | 0 | 2000 | 100 | 0 | 0 |
| 18 | 150 | 3000 | 0 | 190 |  | 0 |
| 19 | 100 | 0 | 700 | 500 | 0 | 1800 |
| 20 | 105 | 2800 | 27000 | 0 | 0 | 0 |
| 21 | 1000 | 60 | 5000 | 2000 | 0 | 0 |
| 22 | 500 | 100 | 0 | 200 | 200 | 0 |
| 23 | 0 | 0 | 20 | 120 | 0 | 0 |
| 24 | 200 | 0 | 0 | 60 | 5000 | 0 |
| 25 | 0 | 100 | 0 | 0 | 0 | 1800 |
| **Total** |  |  |  |  |  |  |
