# Supplementary material for: Assessing the economic impact of climate change in the small-scale aquaculture industry of Ghana, West Africa
Source: AAS Open Res. 2019 Oct 17;1:26. Originally published 2018 Nov 1. [Version 2] doi: 10.12688/aasopenres.12911.2 (PMC7391010; doi:10.12688/aasopenres.12911.2)
Supplement: Supplementary file 5 [file aasopenres-1-14095-s0004.tgz › ee8d16d7-fb9c-4b2c-b6b4-c5f78c989ce2_Supplementary_Table_3.docx]

**Supplementary Table 3: Analyzed climate impact areas**

| **Farm ID** | Climate revenue (₵)  $Cr=\frac{\boldsymbol{CnxNonR}}{\boldsymbol{CP}}$ | Climate cost  $\sum\left( \boldsymbol{Cn} \right)$ | Climate value  $\boldsymbol{Cv=}\frac{\boldsymbol{CrxNonV}}{\boldsymbol{NonR}}$ | Climate weight  $\boldsymbol{Cw=}\frac{\boldsymbol{NonWxCr}}{\boldsymbol{NonR}}$ |
| --- | --- | --- | --- | --- |
| 1 | 67,130.00 | 77000 | 51217.58 | 11178.24 |
| 2 | 24,340.00 | 200 | 32.59984 | 3967.40 |
| 3 | 32,500.00 | 98500 | 15038.17 | 4961.83 |
| 4 | 51,544.00 | 1500 | 158.3591 | 5441.64 |
| 5 | 3,768.00 | 12200 | 1792.792 | 553.71 |
| 6 | 21,650.00 | 0 | 0 | 3500.00 |
| 7 | 18,145.00 | 19930 | 2572.867 | 2342.43 |
| 8 | 8,340.00 | 3000 | 497.3545 | 1382.65 |
| 9 | 1,500.00 | 1100 | 317.3077 | 432.69 |
| 10 | 8,485.00 | 15 | 2.294118 | 1297.71 |
| 11 | 50,030.00 | 40390 | 2635.49 | 3264.51 |
| 12 | 2,650.00 | 2900 | 247.6757 | 226.32 |
| 13 | 3,710.00 | 10700 | 1332.859 | 462.14 |
| 14 | 3,376.00 | 490 | 72.24521 | 497.75 |
| 15 | 497.00 | 8500 | 831.3882 | 48.61 |
| 16 | 3,959.99 | 3000 | 431.0351 | 568.96 |
| 17 | 28,840.00 | 4100 | 537.7049 | 3782.30 |
| 18 | 460.00 | 3340 | 351.5789 | 48.42 |
| 19 | 10,530.00 | 3100 | 568.5987 | 1931.40 |
| 20 | 69,200.00 | 29905 | 2414.005 | 5585.99 |
| 21 | 80,320.00 | 8060 | 1094.365 | 10905.63 |
| 22 | 2,708.00 | 1000 | 176.9148 | 479.09 |
| 23 | 2,160.00 | 140 | 24.34783 | 375.65 |
| 24 | 2,940.00 | 5260 | 416.9512 | 233.05 |
| 25 | 500.00 | 1900 | 427.5 | 112.50 |
| Total |  |  |  | 63580.64 |

**Cr= climate revenue; CP= non-climate total cost of production; Cn = cost of an individual climatic disaster; NonR= non-climate revenue; NonV= non-climate value; Cw= climate weight; and NonW =non-climate weight**
