# Supplementary material for: Assessing the economic impact of climate change in the small-scale aquaculture industry of Ghana, West Africa
Source: AAS Open Res. 2019 Oct 17;1:26. Originally published 2018 Nov 1. [Version 2] doi: 10.12688/aasopenres.12911.2 (PMC7391010; doi:10.12688/aasopenres.12911.2)
Supplement: Peer review history

# Peer review: Reviewer response for version 2

**DOI:** 10.21956/aasopenres.14095.r27222

**Published:** 2020-07-29

**Authors:** Benjamin Apraku Gyampoh

**Affiliations:** 1 Department of Fisheries and Watershed Management, Kwame Nkrumah University of Science and Technology, Kumasi, Ghana

Comments have been addressed.

Is the work clearly and accurately presented and does it cite the current literature?

Partly

If applicable, is the statistical analysis and its interpretation appropriate?

Not applicable

Are all the source data underlying the results available to ensure full reproducibility?

Yes

Is the study design appropriate and is the work technically sound?

Partly

Are the conclusions drawn adequately supported by the results?

Partly

Are sufficient details of methods and analysis provided to allow replication by others?

Yes

Reviewer Expertise:

Climate Change Vulnerability Analysis; Watershed Management; Water Resources Management; Climate Change Adaptation and Mitigation Strategies; Fisheries Management

I confirm that I have read this submission and believe that I have an appropriate level of expertise to confirm that it is of an acceptable scientific standard.

# Peer review: Reviewer response for version 2

**DOI:** 10.21956/aasopenres.14095.r27223

**Published:** 2019-11-26

**Authors:** Anu Heikkilä

**Affiliations:** 1 Finnish Meteorological Institute (FMI) , Helsinki, Finland

The response of the authors to the reviewer's report is mainly satisfactory, but I would like to repeat the request on including a brief discussion on the non-separability of the effects of natural climate variability and climate change in this study. I could not identify a point in the manuscript where the authors would have done this. Furthermore, their response did not indicate that they had taken this request of the reviewer into account.

Is the work clearly and accurately presented and does it cite the current literature?

Partly

If applicable, is the statistical analysis and its interpretation appropriate?

Not applicable

Are all the source data underlying the results available to ensure full reproducibility?

Yes

Is the study design appropriate and is the work technically sound?

Yes

Are the conclusions drawn adequately supported by the results?

Partly

Are sufficient details of methods and analysis provided to allow replication by others?

Yes

Reviewer Expertise:

NA

I confirm that I have read this submission and believe that I have an appropriate level of expertise to confirm that it is of an acceptable scientific standard.

# Peer review: Reviewer response for version 1

**DOI:** 10.21956/aasopenres.13981.r26816

**Published:** 2019-04-03

**Authors:** Anu Heikkilä

**Affiliations:** 1 Finnish Meteorological Institute (FMI) , Helsinki, Finland

**General statement:**

 Asiedu *et al.* report on a study focusing on assessing the multi-faceted effects of climate change on the fish farming industry in Ghana. The metrics they use is the occurrence of events of extreme temperatures, rainfalls, floods, erosion, drought, and storms, and the connection of these occurrences to the decline in the revenues of the farmers.

 The study is important as it brings the problem close to those whose livelihood is directly connected to pond aquaculture and who therefore are in a vulnerable position as the climate change proceeds. The study incorporates questionnaires to the farmers themselves. This has given the voice to a group of people that are already being affected by climate change. The events of flooding, drought, erratic rainfalls and extreme temperatures experienced by the farmers are of course caused by both natural climate variability and climate change. However, separation of these would be a challenging task and would require an independent source of historical climate data.

**Specific comment:**

 It is not quite clear from the beginning of the article how the authors conclude the events of flooding, drought, etc. being caused by climate change. In the questionnaire based approach adopted by the authors the decision on that appears to remain with the farmers. It is rather questionable how they are able to distinguish which events are caused by climate change and which are just natural climatic variability. Would it be possible to normalize the data in any way to separate the actual impact of climate change, using independent historical climate data? The reader would appreciate if the authors could discuss this with a few sentences. The effect experienced by the farmers in their livelihood is all the same, despite the origin of the event causing a decline in their revenue, but the methods and metrics adopted in the study does not allow attributing the effects solely to the climate change.

**Minor remarks:**

1. “The severely of climate change” should be changed to “The severity of climate change”.
2. What is on the x-axis (ranging from 1 to 25) on the graph of Figure 2? Please include a label.
3. “lives people” should be changed to “lives of people”.

Is the work clearly and accurately presented and does it cite the current literature?

Partly

If applicable, is the statistical analysis and its interpretation appropriate?

Not applicable

Are all the source data underlying the results available to ensure full reproducibility?

Yes

Is the study design appropriate and is the work technically sound?

Yes

Are the conclusions drawn adequately supported by the results?

Partly

Are sufficient details of methods and analysis provided to allow replication by others?

Yes

Reviewer Expertise:

atmospheric sciences, atmospheric radiative transfer, interactive effects of ozone depletion, solar UV radiation and climate change

I confirm that I have read this submission and believe that I have an appropriate level of expertise to confirm that it is of an acceptable scientific standard, however I have significant reservations, as outlined above.

# Response

**Published:** 2019-09-15

**Authors:** Berchie Asiedu

**Affiliations:** University of Energy and Natural Resources, Ghana

The main objective was to analyze the impact of climate change using farmers’ perception and farm economics data. The authors agree that the parameters used in the study could be caused by natural climate variability or could be the result of climate change. The present study did not take historical climate data that permit the separation of climate change and natural climate variability. The authors wish to state that it could still be arguable whether the parameters used are the attributable climate change or natural variability of climate even if historical climate data is obtained. Because we did not put much emphasis on time scale and rate of variation of these events. It is on this background that, the study did not put much emphasis on separating natural variability of climate and climate change. We, however, consider the parameters as climate disasters.

**Minor errors:**

1. “severely of climate change” changed to “severity of climate change”. *Correction done.*
2. The x-axis will be labelled appropriately. *Correction done.*
3. “lives people” changed to “lives of people”. *Correction done.*

# Peer review: Reviewer response for version 1

**DOI:** 10.21956/aasopenres.13981.r26670

**Published:** 2018-12-27

**Authors:** Benjamin Apraku Gyampoh

**Affiliations:** 1 Department of Fisheries and Watershed Management, Kwame Nkrumah University of Science and Technology, Kumasi, Ghana

**General Comments**

 1. This is a good paper in a field that is relatively less-researched in Ghana. It is well written. The findings are important baseline to trigger further studies in Ghana on the impacts of climate change on aquaculture production. 

 2. There are some inherent assumptions mostly in the area of cost of climate change impact which is not clear.  It is generally acknowledged that climatic changes would have impacts on climate-dependent activities, both positive and negative. This work focuses on one side – the negative economic impacts. That notwithstanding, the relationships between climatic change and the cost elements of production are not clearly established. 

 3. The authors use three main nodes in their assessment of the economic impact of climate change on small-scale aquaculture industry of Ghana. These are a) operating cost: feed, fingerling, transport, harvest, and labour, b) outputs: total fish weight and value, and c) the incidence of floods, storms, temperature, droughts, rainfall, and erosion and their impacts on farm revenue and maintenance. I think the work would have benefitted from either a Life Cycle Assessment (LCA) or full Value Chain Analysis (VCA) of the selected small-scale fish farms and then subject the LCA or VCA to a climate change vulnerability assessment. This would be a more holistic approach.

**Introduction**

 1. Is it climate change that this work focuses on or is it natural climate variability needs to be set clearly in the introduction. The title clearly states it is the impact of climate change in the small-scale aquaculture industry in Ghana which is the focus. This is not clear in the introduction. Climatic variables can fluctuate without causing the long-term average to change and that refers to the natural variability of climate. When there is entrenched long-term change in the climatic conditions then that brings in climate change. So, “Variability” and “Change” as used in this paper could create confusion as to where the actual focus lies. 

 2. Sieving out certain phenomena or impacts and attributing to climate change or climate variability has become blurred as a result of deliberate or unconscious interchanging use of the terms. 

**Methodology**

 1. The authors explain that a purposive sampling technique was used to select two regions (Ashanti and Brong-Ahafo) out of ten regions in Ghana. They justify selection of the two regions with the statements that majority of small-scale aquaculture (ponds) in Ghana are concentrated in Brong-Ahafo and Ashanti regions and the regions are the core of small-scale aquaculture practices. Quantitative justification would be better. For example, what does ‘majority’ specifically mean? The two regions are from 10 regions in Ghana so good quantitative justification in terms of production percentage would put the justification in proper perspective. 

 2. The Literature used to justify the selection of the Ashanti and Brong Ahafo regions for the study are quite old and will benefit from some newer sources. 

 3. Why a total of 30 farmers were interviewed will need to be explained to establish that number is representative of the aquaculture industry in Ghana. Authors make known that there are about 2600 fish farms in the region. How many of these 2600 farms are owned by the 30 farmers? Or what percentage of total fish production from ponds in the two regions are from these 30 farmers that make them a representative sample? 

**Results and Discussion**

 1. The explanation of climate revenue as the difference between actual revenue (non-climate revenue) and total cost of climatic impact has some inherent assumptions that is difficult to stand. How were the estimation of costs of production accurately attributed to climate change? The issue of correlation, causation and attribution are critical in this situation.  

 2. In figure 5, the authors present climate related disasters at fish farms and the main ‘disasters’ used are flood, temperature, rainfall, drought, and erosion. Flooding of farms, lack of water in ponds and erosion could be as a result of many other watershed factors. Human activities leading to various forms of environmental degradation could lead to ‘droughts’, flooding and erosion and not solely an effect of climate change. Noting from the paper that flood is the most dominant climatic factor of significant economic impact on small-scale aquaculture (Figure 6) throws the debate wide open because the factors that could lead to flooding can be both man-made and natural. In the context of Ghana, some possible causes of flooding include lack of vegetation, land use and land cover changes, development of infrastructure blocking water ways, topography and drainage of the land, poor location of the fish ponds and many more which are more human-induced than climatic. 

 3. The levels of attribution of issues to climate change are quite high in this paper. Should there have been recorded climate data (rainfall, temperature) to support some of the linkages, there would have been good explanation to some extent.

**Conclusion**

 Conclusions are general. Good for planning and future studies.

**References**

 They are OK.

Is the work clearly and accurately presented and does it cite the current literature?

Partly

If applicable, is the statistical analysis and its interpretation appropriate?

Not applicable

Are all the source data underlying the results available to ensure full reproducibility?

Yes

Is the study design appropriate and is the work technically sound?

Partly

Are the conclusions drawn adequately supported by the results?

Partly

Are sufficient details of methods and analysis provided to allow replication by others?

Yes

Reviewer Expertise:

Climate Change Vulnerability Analysis; Watershed Management; Water Resources Management; Climate Change Adaptation and Mitigation Strategies; Fisheries Management

I confirm that I have read this submission and believe that I have an appropriate level of expertise to confirm that it is of an acceptable scientific standard, however I have significant reservations, as outlined above.

# Response

**Published:** 2019-01-31

**Authors:** Berchie Asiedu

**Affiliations:** University of Energy and Natural Resources, Ghana

**Introduction**

1. Yes, the focus is on climate change. Climate change is the result of natural climate variation and anthropogenic climate effects. Natural climate variability is used here as an aspect of climate change, not a substitute. Climatic variables could fluctuate and cause significant variation climate. So, using climate variability in the context of climate change should not be strange.

**Methodology**

         

1. Suggestion: The area has about 2,600 fish ponds making up about 55 percent of the total ponds in Ghana (MoFAD, 2014). Pond aquaculture abundance was the criteria used in site selection. In terms of national pond production, the two regions account for 40 percent of the pond production (MoFAD, 2012).
2. The authors agreed that the literature used for the selection of the Ashanti region is quite old. However, the documents provide important description of the selected site.
3. Not 2,600 fish farms, but 2,600 fish ponds. Registered farm list was obtained from the Fisheries Commission. There were 70 and 50 farms in Ashanti region and Brong-Ahafo region, respectively. This makes up a total of 120 farms. Authors sampled 25 percent (i.e. 30 farms) of the total farm list obtained for the farm level interview.

**Results and Dis** **cussion**

 1. Kindly see sample questionnaire and supplementary Table 2 for further clarity. Firstly, incidence of climate disasters at farms was obtained. And when farms experienced these disasters what is the cost? These include fish mortalities (value loss), stock loss under severe floods (value), cost of fighting erosion and maintaining pond dikes, cost of pumping water in drought conditions, and cost destroyed farm structures by climate disasters. 

 2. Used climate disasters are weather events. Arguably, these could be the result of many watershed factors or human activities. Human activities impact both climate variation and watershed factors. Whilst changes in climate impacts watershed, which could result in floods, droughts, etc. Yes, flooding maybe both man-made or natural. That is why climate change looks at both internal processes (natural climate variability) or external forces (persistent anthropogenic changes in land use, vegetation cover, etc). 

 3. The climate disasters defined by this study are projected to impact ways by which climate change will affect the fisheries and aquaculture inustry ((Allison et al., 2005; WordFish, 2007; De Silva & Soto, 2009; Yazdi & Shakouri, 2010) by many studies. The author assessed the impacts of such disasters in small-scale aquaculture industry of Ghana.
